# Chloroquine for Covid 19: introducing drug repurposing to medical students

**DOI:** 10.5116/ijme.5f09.79e2

**Published:** 2020-07-24

**Authors:** Gideon Koren, Liat Korn

**Affiliations:** 1 Adelson School of Medicine, Ariel University, Ariel, Israel; 2 Faculty of Health Sciences, Ariel University, Ariel, Israel

**Keywords:** Chloroquine, Covid 19, drug repurposing, medical students

## To the Editor

The teaching of clinical pharmacology concepts to medical students can often be complex and challenging, resulting in inadequate preparation for clinical practice.^1^  The concepts of drug repurposing, the use of an existing drug for a new indication, are relatively new to medical school curricula. The recent attempts to manage the Covid 19 infection with an old drug, chloroquine,^2^ provides a unique learning opportunity to introduce these concepts to medical students. We hypothesized that the pandemic nature of the infection and the tremendous efforts to identify medications to treat this condition will render this topic more relevant to medical students than learning from historical examples. We have previously used this approach in introducing the popular movie Awakenings to teaching study design and ethical issues with the use of L Dopa to treat chronic encephalitis, based on the true experience of Dr. Oliver Sacks in New York.^3^ The Awakening module has been used by us since 1994 in several countries and medical schools including the National Institute of Child Health and Human Development summer institute in drug use in pregnancy. We present a learning module dedicated to drug repurposing, involving students’ critical appraisal of selected scientific papers, followed by presentation and group discussion. Each element of the module is introduced here by the basic information the mentor is expected to cover:

Covid 19 has been identified in late 2019 as the cause of the pandemic experienced in virtually every country worldwide.^4^^-^^5^ The disease has caused the death of thousands of people with China, Italy, the UK, the USA and South Korea leading in numbers. While a vaccine may prevent the infection, the process of developing vaccines typically takes years to accomplish.^5^ In the meantime, there is an urgent need to identify drugs that may exert antiviral activity against Covid 19. The development of new antivirals will depend on a deeper understanding of the molecular mechanisms of the virus. Because of the serious health risks of the Covid 19, most notably the acute respiratory distress syndrome, attempts to identify existing medications which may exert antiviral properties are warranted.^6^

The first task in our module involves the students preparing and presenting an update on the Covid 19 pandemic and its management, followed by a group discussion and comments by the mentor. Historically there have been dramatic examples of medications used for one condition being re-purposed for new, unmet indications. For the purpose of this module we selected the anti- depressant drug bupropion (Wellbutrin) which was introduced for depression in the 1980s,^7^  but which, during Phase 3 clinical trials, was shown to decrease the urge for smoking.^7^ This observation had led the manufacturer to study the antismoking effect of bupropion, and subsequently to the introduction of the first oral anti-smoking agent, under the trade name Zyban.^8^

The cost of developing a new drug nearly doubles every nine years, and as of 2016, developing a new medication requires more than 14 years and 2–3 billion dollars in cost.^9^   Given this prohibitive cost, pharmaceutical companies have increased investments in drug repurposing, the process of applying known drugs to treat new diseases. It has been estimated that drug repurposing cuts development time in half and significantly reduces costs.^9^ Other successful examples of repurposing include sildenafil,  aimed originally in 1989 for hypertension and angina, which was subsequently found to be an effective treatment for erectile dysfunction in 1998.^10^ Repurposing efforts may also include re-examination of previously- failing drugs for novel treatments: For example, azidothymidine, originally designed as a chemotherapeutic agent, was repurposed in the 1980s as an anti-HIV drug.^11^  In the case of the present Covid 19 outbreak, the main advantage of chloroquine, if proven to be effective and safe, is the immediacy of its availability.

In improving repurposing efforts, there are now increasing numbers of studies using big data analytics and machine learning techniques to identify whether drugs presently given to patients for proven indications may exert other

therapeutic effects not previously thought of. For example, it has been recently shown, using novel algorithms based on over 2 millions electronic patient records, that statins and proton pump inhibitors decrease blood pressure independently of typical antihypertensive drugs.^12^ Similarly, it has been documented that alpha 2 blockers exert a hypoglycemic effect, independent of the hypoglycemic effects of regular antidiabetic drugs.^13^

The second task involved students presenting the published evidence leading to labeling bupropion as an antismoking agent, followed by group discussion .The identification of repurposable agents against Covid 19 involves a screening process in which different in vitro concentrations of a given drug are examined for their ability to inhibit the viral replication in appropriate cell culture.^14^  The drugs selected to be tested can be included based on preliminary clinical evidence, theoretical mechanistic evidence, or no evidence at all. For example, in searching for an agent that can down-regulate the cerebral production of the alpha-synuclein protein encoded by the alpha-synuclein gene in cases of Parkinson’s disease, Mittal  and colleagues screened 1000 compounds without any hypothetical assumptions, discovering and then proving clinically that beta mimetic agents inhibit such accumulation.^15^

The third task in our module involved students presenting the experiments and dose-response results of chloroquine on Covid 19 virus replication, including the concentrations yielding 50% effective inhibition.^14^ Phase 1 studies aim at defining the safety of a molecule before it can be given to humans. Typically a small group of healthy volunteers receive the drug in escalating doses till an adverse event is encountered. Then the researchers go down to the second-highest dose, and this level will be considered the highest dose to be given to humans.^16^ In the case of chloroquine, its safety profile has been well defined after 70 years of clinical use for malaria and immunological conditions such as lupus erythematosus.^17^ Hence this phase can be theoretically skipped in our case. However, it is possible that the effective anti- viral dose needs to be higher than for the indications it had been used for previously. In that case, the dose-response safety curve needs to be extended.

Task 4 involved students discussing the published evidence on chloroquine safety, based on dose and serum concentrations.^17^^-^^18^ Phase 2 studies are executed either in healthy volunteers or people with the disease in question.  One of the objectives of Phase 2 trial is to characterize the pharmacokinetics of the drug.^16^  This would indicate whether the levels produced in vivo are sufficient in achieving the concentrations shown to be effective in vitro.

Task 5 involved students discussing whether the identified Phase 2 studies allow achievement of safe concentrations of chloroquine for Covid 19 to move forward to Phase 3 studies. Phase 3 studies are the penultimate step toward proving efficacy and safety of a drug against placebo or against the standard of care therapy.^16^  A protocol has to succinctly describe the patient population, inclusion and exclusion criteria, primary and secondary endpoints, the dose and mode of intervention, potential confounders and sample size.

Task 6 involved students describing the outline of the protocol and all steps involved.^19^  The learning module presented here is intended to be an interactive experience where medical students are involved in learning, critical appraisal and group discussion. However, it can also be taught as a frontal lecture in cases where the interactive element cannot be achieved in a big class. In addition, we also hoped to create an opportunity for students to experience how to handle new scenarios of therapeutic challenges not previously encountered. Since the inception of this module, we have executed and tested it in two classes, with excellent engagement and evaluations by the learners. A substantial proportion of students rated it as one of their best learning experiences. While there are increasing numbers of drugs with more than one proven indication, new approaches to identify re- purposable drugs will be part of tomorrow’s reality for today’s medical students.

### Conflict of Interest

The authors declare that they have no conflict of interest.

## References

[1] Keijsers CJ, van Hensbergen L, Jacobs L, Brouwers JR, de Wildt DJ, ten Cate OT, Jansen PA (2012). Geriatric pharmacology and pharmacotherapy education for health professionals and students: a systematic review.. Br J Clin Pharmacol.

[2] Touret F, de Lamballerie X (2020). Of chloroquine and COVID-19.. Antiviral Res.

[3] Koren G (1993). Awakenings: using a popular movie to teach clinical pharmacology.. Clin Pharmacol Ther.

[4] Bhatraju PK, Ghassemieh BJ, Nichols M, Kim R, Jerome KR, Nalla AK, Greninger AL, Pipavath S, Wurfel MM, Evans L, Kritek PA, West TE, Luks A, Gerbino A, Dale CR, Goldman JD, O'Mahony S, Mikacenic C (2020). Covid-19 in Critically Ill Patients in the Seattle Region - Case Series.. N Engl J Med.

[5] Kormann C. How long will it take to develop a Corona virus vaccine? 2020. [Cited 08 March 2020] Available from: https://www.newyorker.com/news/news-desk/how-long-will-it-take-to-develop-a-coronavirus-vaccine www.newyorker.com.

[6] Jones DS (2020). History in a Crisis - Lessons for Covid-19.. N Engl J Med.

[7] Stern WC, Harto-Truax N (1980). Two multicenter studies of the antidepressant effects of bupropion HCl versus placebo.. Psychopharmacol Bull.

[8] Hughes JR, Goldstein MG, Hurt RD, Shiffman S (1999). Recent advances in the pharmacotherapy of smoking.. JAMA.

[9] Nosengo N (2016). Can you teach old drugs new tricks?. Nature.

[10] Boolell M, Gepi-Attee S, Gingell JC, Allen MJ (1996). Sildenafil, a novel effective oral therapy for male erectile dysfunction.. Br J Urol.

[11] Oksenhendler E (1989). Azidothymidine.. Nouv Rev Fr Hematol.

[12] Koren G, Nordon G, Radinsky K, Shalev V (2018). Machine learning of big data in gaining insight into successful treatment of hypertension.. Pharmacol Res Perspect.

[13] Koren G, Nordon G, Radinsky K, Shalev V (2019). Identification of repurposable drugs with beneficial effects on glucose control in type 2 diabetes using machine learning.. Pharmacol Res Perspect.

[14] Keyaerts E, Li S, Vijgen L, Rysman E, Verbeeck J, Van Ranst M, Maes P (2009). Antiviral activity of chloroquine against human coronavirus OC43 infection in newborn mice.. Antimicrob Agents Chemother.

[15] Mittal S, Bjørnevik K, Im DS, Flierl A, Dong X, Locascio JJ, Abo KM, Long E, Jin M, Xu B, Xiang YK, Rochet JC, Engeland A, Rizzu P, Heutink P, Bartels T, Selkoe DJ, Caldarone BJ, Glicksman MA, Khurana V, Schüle B, Park DS, Riise T, Scherzer CR (2017). β2-Adrenoreceptor is a regulator of the α-synuclein gene driving risk of Parkinson's disease.. Science.

[16] American cancer society. What are the phases of clinical trials? 2020. [Cited 14 March 2020]; Available from: https://www.cancer.org/treatment/treatments-and-side-effects/clinical-trials/what-you-need-to-know/phases-of-clinical-trials.html.

[17] Naing C, Aung K, Win DK, Wah MJ (2010). Efficacy and safety of chloroquine for treatment in patients with uncomplicated Plasmodium vivax infections in endemic countries.. Trans R Soc Trop Med Hyg.

[18] Rainsford KD, Parke AL, Clifford-Rashotte M, Kean WF (2015). Therapy and pharmacological properties of hydroxychloroquine and chloroquine in treatment of systemic lupus erythematosus, rheumatoid arthritis and related diseases.. Inflammopharmacology.

[19] Stanley K (2007). Design of Randomized Controlled Trials.. Circulation.

